# The Interplay Between Stress Related Genes and Its Role in Human Longevity: Insights for Translational Studies

**DOI:** 10.1093/geroni/igab046.2519

**Published:** 2021-12-17

**Authors:** Anatoliy Yashin, Deqing Wu, Konstantin Arbeev, Olivia Bagley, Igor Akushevich, Arseniy Yashkin, Matt Duan, Svetlana Ukraintseva

**Affiliations:** 1 Duke University, Durham, North Carolina, United States; 2 Duke University, Morrisville, North Carolina, United States

## Abstract

Human lifespan is a multifactorial trait resulted from complicated interplay among many genetic and environmental factors. Despite substantial progress in clarifying many aspects of lifespan’ variability the mechanism of its multifactorial regulation remains unclear. In this paper we investigate the role of genes from integrated stress response (ISR) pathway in such regulation. Experimental studies showed that persistent cellular stress may result in cellular senescence (for proliferating cells), or in apoptosis (for post-mitotic cells) which may affect health and lifespan in laboratory animals. These studies also showed which ISR genes are likely to interplay to produce joint effects on these traits. Note that in humans, the interplay between these genes does not necessarily influence these traits. This is because biological mechanisms regulating these traits in laboratory animals and humans may differ. This means that, when possible, the experimentally detected connections promising for human applications, should be verified using available human data before their testing in expensive clinical trials. In this paper we used HRS data to test connection between SNPs from the EIF2AK4 gene that senses cellular stress signals and the DDIT3 gene from the apoptosis regulation part of the ISR. We found genome wide significant associations between interacting SNPs from these genes and longevity. This result shows that available human data may be successfully used for making important steps in translation of experimental research findings towards their application in humans. Following this strategy may increase efficiency of clinical trials aiming to find appropriate medications to promote human health and longevity.

